# Knockdown of AQP1 inhibits growth and invasion of human ovarian cancer cells

**DOI:** 10.3892/mmr.2021.12503

**Published:** 2021-10-19

**Authors:** Yanli Wang, Yanyan Fan, Chunhua Zheng, Xiaomeng Zhang

Mol Med Rep 16: 5499-5504, 2017; DOI: 10.3892/mmr.2017.7282

Following the publication of this paper, it was drawn to the Editors’ attention by a concerned reader that certain of the western blotting data shown in Fig. 2B and flow cytometric data shown in Fig. 3B bore unexpected similarities to data appearing in different form in other articles by different authors. Furthermore, there were additional concerns regarding the possibly anomalous appearance of cell migration data shown in Fig. 4B. Owing to the fact that some of the contentious data in the above article had already been published elsewhere, or were already under consideration for publication, prior to its submission to *Molecular Medicine Reports*, the Editor has decided that this paper should be retracted from the Journal. After having been in contact with the authors, they agreed with the decision to retract the paper. The Editor apologizes to the readership for any inconvenience caused.

